# Coil embolization of an anomalous bronchial artery originating from the left subclavian artery following arterial switch operation: a case report

**DOI:** 10.1186/s13256-015-0540-9

**Published:** 2015-03-08

**Authors:** Edvin Prifti, Fadil Ademaj, Arben Baboci, Efrosina Kajo, Vittorio Vanini

**Affiliations:** 1 Cardiac Surgery Department, Tirana University Medical Center, Tirana, Albania; 2 Heart Disease Department, Gjakova Hospital, Rr Prizren, Gjakove, Kosovo; 3 Children’s Heart Foundation, Bergamo, Italy

**Keywords:** Anomalous bronchial artery, Arterial switch operation, Coil embolization, Transposition of the great arteries

## Abstract

**Introduction:**

Bronchial arteries originate from the descending aorta at the level of the T5-T6 vertebrae following an intrapulmonary course along the major bronchi. When bronchial arteries take off from a vessel other than the descending aorta, the anatomy is defined as an anomalous origin of the bronchial artery.

**Case presentation:**

A 3-day-old boy from Kosovo with dextro-transposition of the great arteries who developed progressive heart failure required an emergency arterial switch operation. Because of persistent pulmonary edema after completion of the arterial switch operation at our institution, the patient could not be weaned off mechanical ventilation. Transthoracic echocardiography revealed an anomalous accelerated flow, indicating an anomalous systemic pulmonary shunt. Arterial catheterization revealed an abnormal bronchial artery originating from the left subclavian artery and bifurcating to both lungs. The anomalous ectatic bronchial artery was successfully occluded by coil embolization. The improvement of the patient’s hemodynamic status resulted in an uneventful post-operative course.

**Conclusion:**

A coil embolization procedure was successfully performed to treat an anomalous bronchial artery originating from the left subclavian artery after a switch operation in a patient with transposition of the great arteries. When clinically indicated, catheter-based therapy with coil embolization can be performed to successfully treat anomalous bronchial arteries by reducing as such the pulmonary overflow.

## Introduction

Bronchial arteries (BAs) normally originate from the descending thoracic aorta at the level of the T5-T6 vertebrae with an intrapulmonary course along the major bronchi [1]. Anomalous origin of a BA is defined as a vessel that arises outside these areas [2]. These arteries can be identified in the fetus as early as 9 weeks of gestational age, and they are usually quite small and hemodynamically insignificant. In 1863, Cockle [3] described the first case in the literature of enlarged BA in association with transposition of the great arteries (TGAs) discovered in a patient post-mortem. These vessels have been implicated in the pathogenesis of accelerated pulmonary vascular disease, and, in some cases, they may produce a volume overload to the systemic ventricle. In this report, we describe a case of a pediatric patient with dextro-TGA (d-TGA) of anomalous BA origin and distribution who developed hypoxemia, cardiac insufficiency and pulmonary edema after undergoing an arterial switch operation (ASO). He was successfully treated using a coil embolization procedure to close the anomalous BA.

## Case presentation

**Figure 1:**
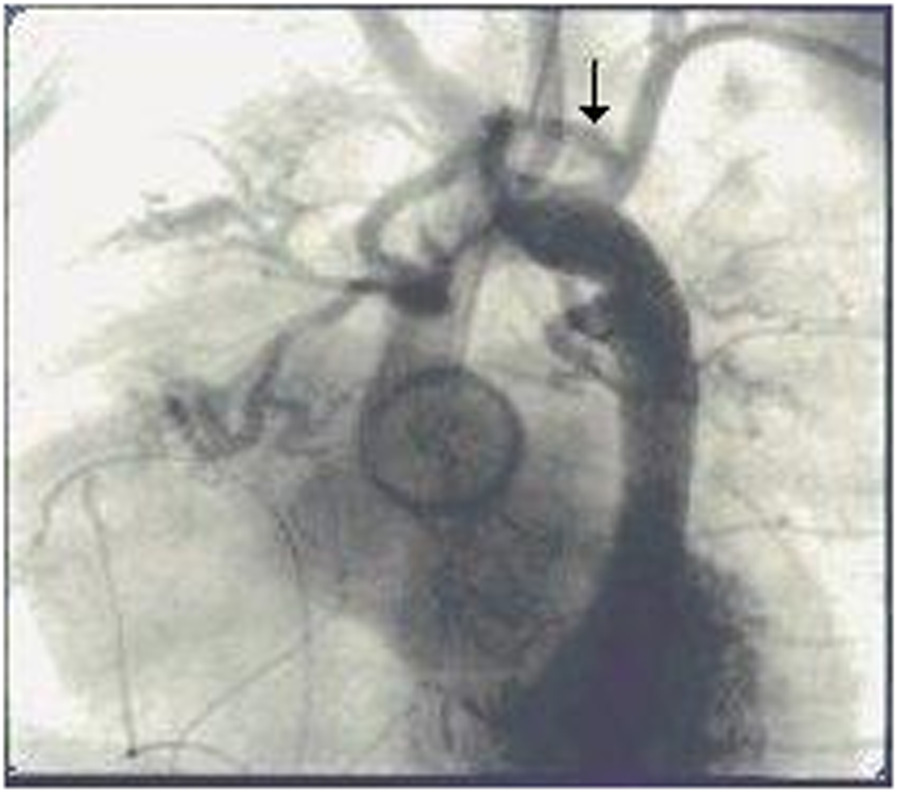
**The arrow indicates an abnormal bronchial artery arising from the inferior wall of the left subclavian artery, from which it divides into two branches that give flow to both lungs as bronchial arteries.**

**Figure 2:**
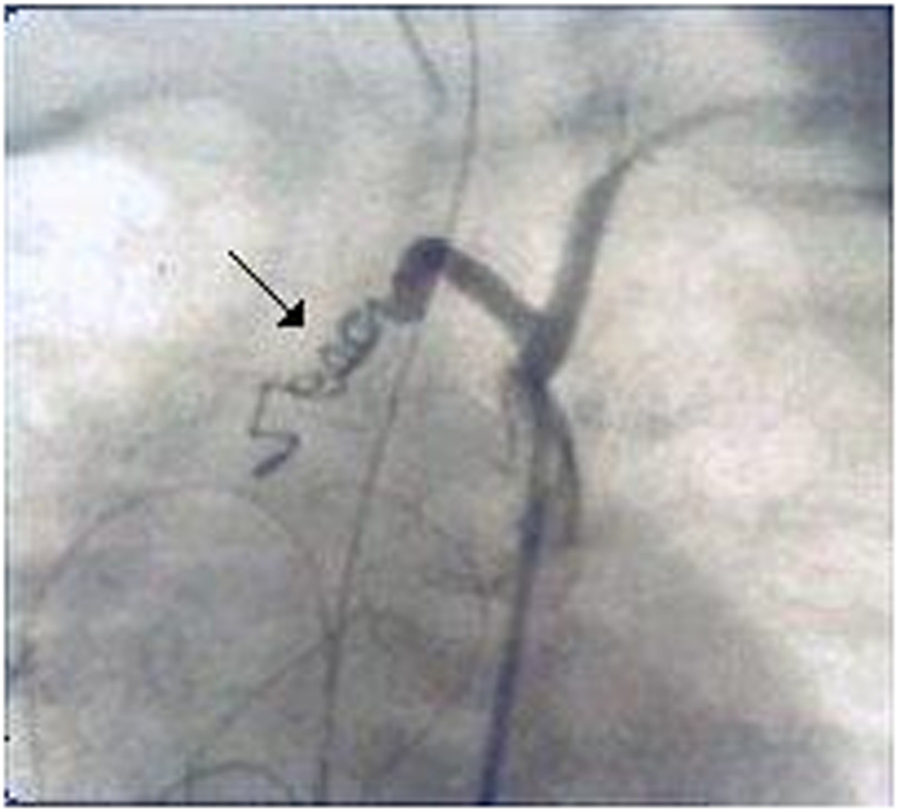
**The arrow indicates the successful closure with coil embolization of the abnormal bronchial artery before its bifurcation.**

A 3-day-old boy from Kosovo, weighing 2.4kg, was admitted to our hospital with severe dyspnea and cyanosis. Transthoracic echocardiography revealed d-TGA with intact ventricular septum, left aortic arch and a 4mm patent ductus arteriosus. Medical therapy with prostaglandin E_1_, dopamine, oxygen and antibiotics was initiated. The following day, owing to hemodynamic instability, urgent corrective cardiac surgery was necessitated. The intra-operative findings included the following. The sinus 2, according to the Leiden convention [4], presented two coronary ostia: one giving off a branch as the right coronary artery, and the other one originating at a long left main coronary trunk and passing directly backward in close relation to the pulmonary artery, then curving around its posterior aspect to reach the atrioventricular groove, from whence it bifurcated into the left anterior descending and circumflex arteries. An additional branch originated from sinus 1 as a conal branch, giving rise to the septal branches. Translocation of the coronary arteries was performed. The new pulmonary trunk was reconstructed to avoid compression of the coronary artery and distortion of the great arteries. Immediately after surgery, the patient was unstable hemodynamically, as demonstrated by left ventricular (LV) failure and persistent pulmonary edema requiring inotropic support and mechanical ventilation. Transthoracic echocardiography revealed the presence of an anomalous accelerated Doppler flow localized at the left side of the heart, precisely at the aortic arch level. This Doppler image gave rise to our suspicion of an anomalous shunt between the systemic and pulmonary circulation. The hemodynamic study demonstrated a unique supra-aortic anomalous vessel originating from the left subclavian artery. The anteroposterior projection demonstrated an anomalous vessel originating from the inferior wall of the left subclavian artery, from whence it divided into two branches supplying both lungs as BAs (Figure 1). The anomalous BA originating from the left subclavian artery was the principal cause of a pulmonary hyperperfusion with consequent pulmonary edema and congestive heart failure. There were no BA originating from the T5-T6 segments. The anomalous vessel was successfully occluded proximally to its bifurcation in the catheterization laboratory with a 5-French catheter (Terumo Europe, Leuven, Belgium) and two Gianturco coils 3×3-French (William Cook Europe, Bjæverskov, Denmark) inserted through the left subclavian artery (Figure 2). The closure of this anomalous systemic pulmonary communication was associated with significant improvement of the hemodynamic and clinical status of the newborn, permitting successful extubation and decrease of the inotropic support. The patient’s post-operative course was uneventful, and the baby was discharged to home 30 days later in excellent clinical condition.

## Discussion

The most common complications after an ASO are the supravalvular pulmonary stenosis, neoaortic valve dysfunction and myocardial ischemia related to coronary artery translocation [5,6], such as obstruction of coronary arteries commonly caused by the development of fibrous tissue at the coronary button suture lines or vessel “kinking” secondary to an imperfect arterial translocation [7]. Several other factors can complicate the post-operative course, such as the presence of abnormal and enlarged BAs, which is associated with increased pulmonary blood flow and LV load as well as a low survival rate [8].

Hypoxemic respiratory failure happens as a result of atelectasis, infection, pulmonary immaturity or surfactant deficiency, pulmonary hypertension and ventilation/perfusion mismatch. The presence of a left-to-right shunt due to enlarged BAs might be another cause of respiratory failure [9]. In patients with TGA undergoing ASO, the lungs are congested because of increased blood flow through these collateral arteries, which causes pulmonary edema and congestive heart failure.

About half of patients with TGA undergoing ASO present with anomalous bronchial collateral arteries detected during post-operative cardiac catheterization studies [8]. Flow through these BAs is trivial to mild in the majority of patients and appears to be of no hemodynamic significance, but a few patients have significant LV volume load due to enlarged BAs [8]. Evidence of cardiomegaly, congestive heart failure due to increased LV end-diastolic pressure and pulmonary arterial hypertension following ASO may indicate an increased pulmonary blood flow. Such a condition should be differentiated from other causes of LV failure after ASO, such as myocardial ischemia or infarction due to coronary obstruction after the translocation, neoaortic valve insufficiency or myocardial protection during surgery [5].

Abnormally enlarged BAs might not be diagnosed in patients with simple TGA, as pre-operative angiography is rarely performed. Unexplained pulmonary hypoxemia with pulmonary hypercirculation after ASO could be an indicator of anomalous enlarged BAs. Post-operative cardiac catheterization permits the clinician to visualize the pulmonary veins and left atrium after injection of contrast medium into the aorta. The presence of enlarged BAs should be differentiated from the major aortopulmonary collateral arteries (MAPCAs), which supply blood to the lungs when the native pulmonary circulation is underdeveloped, which carries a pathophysiology different from simple TGA. The MAPCAs’ sites of origin are from the descending aorta (70% of cases), aortic arch (15% to 20% of cases) and ascending aorta (10% to 15% of cases) [10].

Previous studies have documented enlarged BAs in patients with TGA [6,9]; however, significant bronchial collateral arteries complicating early post-operative recovery following neonatal ASO are extremely rare. Irving and colleagues [9] presented a case of a patient with enlarged BA complicating the early post-operative course after an ASO in a newborn, requiring coil embolization.

The most interesting finding in our patient was the anomalous origin of a single-trunk BA originating from the left subclavian artery that furnished both lungs simultaneously. Such an abnormality is scarcely reported in the literature [11,12] and has never been reported in combination with the TGA. Sancho *et al.* [2] demonstrated that 27 (8.3%) patients had an anomalous origin of BA in a series of 300 BAs undergoing embolization, and in only one did it originate from the right subclavian artery. Other anomalous origins are the aortic arch, thyrocervical trunk, lower descending thoracic aorta, costocervical trunk, brachiocephalic artery, pericardiophrenic artery, inferior phrenic artery, abdominal aorta and coronary artery [13].

Late surgical ligation or coil embolization of these collateral arteries has been reported following ASO [14]. Although the experience is very limited, the coil embolization procedure leads to excellent outcomes in this case [2,9,15]. Coil embolization has been employed successfully even in cases with an anomalous origin of the BA from the right subclavian artery [12], but until now it has not been reported in association with TGA. This case report demonstrates that such intervention after ASO is feasible and offers the prospect of an excellent outcome, even when the BA originates abnormally from the left subclavian artery.

## Conclusions

In this report, we describe a case of a pediatric patient with a combination of an anomalous BA originating from the left subclavian artery and TGA, whom we treated successfully with coil embolization. Catheter-based therapy with coil embolization can be performed with good results when clinically indicated, reducing pulmonary overperfusion and avoiding the risks of a surgical procedure.

## Consent

Written informed consent was obtained from the patient's legal guardian for publication of this case report and any accompanying images. A copy of the written consent is available for review by the Editor-in-Chief of this journal.

## Abbreviations

ASO: Arterial switch operation
BA: Bronchial artery
LV: Left ventricular
MAPCA: Major aortopulmonary collateral artery
TGA: Transposition of the great arteries
